# Spatio-temporal heterogeneity and driving mechanisms of RSEI in the north-south sections of the Beijing-Hangzhou grand canal: an empirical study using GEE and XGBoost-SHAP

**DOI:** 10.1038/s41598-026-45721-9

**Published:** 2026-04-09

**Authors:** Xiaoli Xia, Shangpeng Sun, Qiao Liu, Hui Guo, Yuanbing Wang

**Affiliations:** 1 Zaozhuang University, Zaozhuang, China; 2 Anhui Meteorological Service, Hefei, China; 3 Nanjing Agricultural University, Nanjing, China; 4 Nanjing University of Information Science and Technology, Nanjing, China

**Keywords:** Remote sensing ecological index (RSEI), Beijing-Hangzhou grand canal, Google earth engine (GEE), XGBoost, SHAP, Driving mechanism, Ecology, Ecology, Environmental sciences

## Abstract

The Beijing-Hangzhou Grand Canal, a vital ecological corridor and cultural heritage site, requires a comprehensive understanding of the spatio-temporal evolution and driving mechanisms of its ecological environment to support sustainable regional development. This study leveraged the Google Earth Engine cloud platform and MODIS growing-season imagery (May-September, 2000–2020) to assess the spatiotemporal dynamics of ecological quality along the entire canal using the Remote Sensing Ecological Index (RSEI). An explainable machine learning framework (XGBoost-SHAP) was further applied to quantitatively disentangle the contributions of natural and anthropogenic drivers underlying the observed spatial heterogeneity in RSEI. The results revealed that: (1) A pronounced and persistent north-south gradient in RSEI values was identified, with ecological quality consistently higher in southern regions compared to northern regions over the two-decade period; and (2) the driving mechanisms demonstrated distinct differences between sections-the ecological quality in the northern section was primarily shaped by natural factors such as precipitation and temperature (“natural factor-dominated” regime), whereas in the southern section it was mainly driven by nighttime light intensity, indicative of urbanization (human activity-dominated” regime). This study elucidates the differential causes of ecological quality divergence between the north and south sections of the canal. The integrated GEE and XGBoost-SHAP framework provides a robust and interpretable approach for attribution analysis in complex environmental systems. This approach has the potential to be extended to other large linear ecosystems and provides a scientific basis for region-specific ecological protection and restoration strategies.

## Introduction

The Beijing-Hangzhou Grand Canal, the world’s longest artificial waterway, is a vital ecological corridor and cultural heritage artery connecting northern and southern China^1^. Its functions in navigation, irrigation, and ecosystem regulation are crucial for regional sustainability^2,3^. However, rapid urbanization and industrialization have subjected its ecological environment to severe pressures, such as water pollution, habitat loss, and biodiversity decline^4,5^. These challenges necessitate accurate, dynamic monitoring of ecological quality and a mechanistic understanding of its drivers to support effective conservation^6^.

Extending over 1,800 km through diverse climatic and socio-economic zones, the Canal exhibits a pronounced north-south gradient in natural endowments (e.g., precipitation, temperature) and human pressures^7,8^. Although previous research has acknowledged the ecological heterogeneity along the Canal^9–11^, a systematic, basin-wide understanding of its underlying driving mechanisms is still lacking. Previous research has advanced our knowledge through localized assessments, such as habitat quality evaluation in the Suzhou Section^12^, water quality monitoring in Beijing^13^, and vegetation dynamics analysis^14^. However, three critical gaps persist: (1) most studies are confined to specific sections or single ecological elements, lacking a holistic, full-basin perspective; (2) methodologically, they often rely on traditional statistical models (e.g., linear regression, geographic detectors) which struggle to capture the complex nonlinear relationships inherent in coupled human-natural systems^15^; (3) consequently, a quantitative and interpretable attribution of the drivers behind the well-recognized north–south ecological divergence is still inadequate.

Significant efforts have been made to monitor and assess the ecological status of the Grand Canal, with research methodologies continuously evolving. At the full-basin scale, Li et al. pioneered an integrated PCA-AHP-TOPSIS methodology to estimate ecological indices based on remote sensing. It provides a novel paradigm for comprehensive ecological assessment^16^. For specific sections, studies have yielded detailed insights. For instance, Zhang et al. conducted a habitat quality assessment and ecological risk prediction for the Suzhou section, revealing its vulnerability during urbanization and noting a 12.7% decline in habitat quality between 2015 and 2020, primarily driven by construction land expansion^12^. Complementing this, Tang et al. discussed the canal along the spatio-temporal coupling relation between urbanization and habitat quality, identifying an inverted U-shaped relationship that offers a critical theoretical basis for balancing conservation and development^17^. Using satellite data for water quality assessment within the SDG11.4 framework, Xie et al. focused on the Beijing section, and reported a 30% improvement in water transparency since 2015, highlighting successful remediation efforts^13^. Furthermore, Jiang et al. analyzed the spatio-temporal dynamics of vegetation coverage and found that from 2000 to 2020, vegetation coverage generally showed an increasing trend, but there was obvious spatial heterogeneity. The Shandong section showed the most remarkable improvement (18.5%), while parts of southern Jiangsu, constrained by urban expansion, improved by only 6.3%^14^. Despite these advancements, notable research gaps persist. Most studies are confined to specific sections or single ecological elements, lacking a systematic, full-basin comparative analysis. Methodologically, they often rely on traditional statistical models, which have limited capacity to decipher the complex nonlinear relationships inherent in this ecosystem. Crucially, a quantitative and mechanistic understanding of the drivers behind the well-recognized north-south.

Furthermore, the scale of analysis required to address these gaps poses significant computational challenges. Conventional methods relying on desktop-based software and local data storage are often inefficient when processing the large volumes of satellite imagery required for full-basin, multi-decadal assessments^18,19^. This is particularly true for a linear corridor as extensive as the Grand Canal, where traditional approaches would involve cumbersome data download, preprocessing, and mosaicking steps. The emergence of cloud-based platforms, particularly Google Earth Engine (GEE), has revolutionized large-scale environmental monitoring by providing access to a massive petabyte-scale catalog of satellite data and enabling high-performance parallel computing^18^. GEE has been successfully integrated with various analytical models for ecological assessments, ranging from land cover classification and time-series analysis to the calculation of complex indices like the RSEI^20,21^. Its capacity to efficiently handle both the spatial extent (entire canal buffer) and temporal depth (2000–2020) of this study makes it an ideal platform for our data preprocessing and RSEI computation.

To bridge these gaps, this study proposes an integrated analytical framework that couples the Google Earth Engine (GEE) cloud platform with an explainable machine learning framework integrating XGBoost with SHAP (SHapley Additive exPlanations)^22,23^. While XGBoost and SHAP are established methods, their synergistic application within a GEE-based workflow for long-term, full-basin ecological attribution represents a novel paradigm for linear ecosystem studies^24–26^. This framework offers distinct advantages: (1) GEE enables efficient processing of long-term satellite imagery (MODIS, 2000–2020) for basin-wide Remote Sensing Ecological Index (RSEI) assessment; (2) XGBoost effectively models complex, nonlinear relationships between RSEI and its potential drivers; and (3) SHAP provides quantitative, interpretable insights into driver contributions, moving beyond mere correlation to reveal directional impacts and interactions.

Therefore, this study aims to: (1) characterize the spatio-temporal patterns of ecological quality (RSEI) along the entire Canal from 2000 to 2020; and (2) quantify and compare the natural and anthropogenic driving mechanisms underlying the north-south ecological divergence using the XGBoost-SHAP interpretability framework. By doing so, we seek to establish a scientific basis for spatially differentiated ecological management and demonstrate a transferable methodology for assessing large-scale linear heritage corridors worldwide.

## Data and methodology

### Study area

**Fig. 1:**
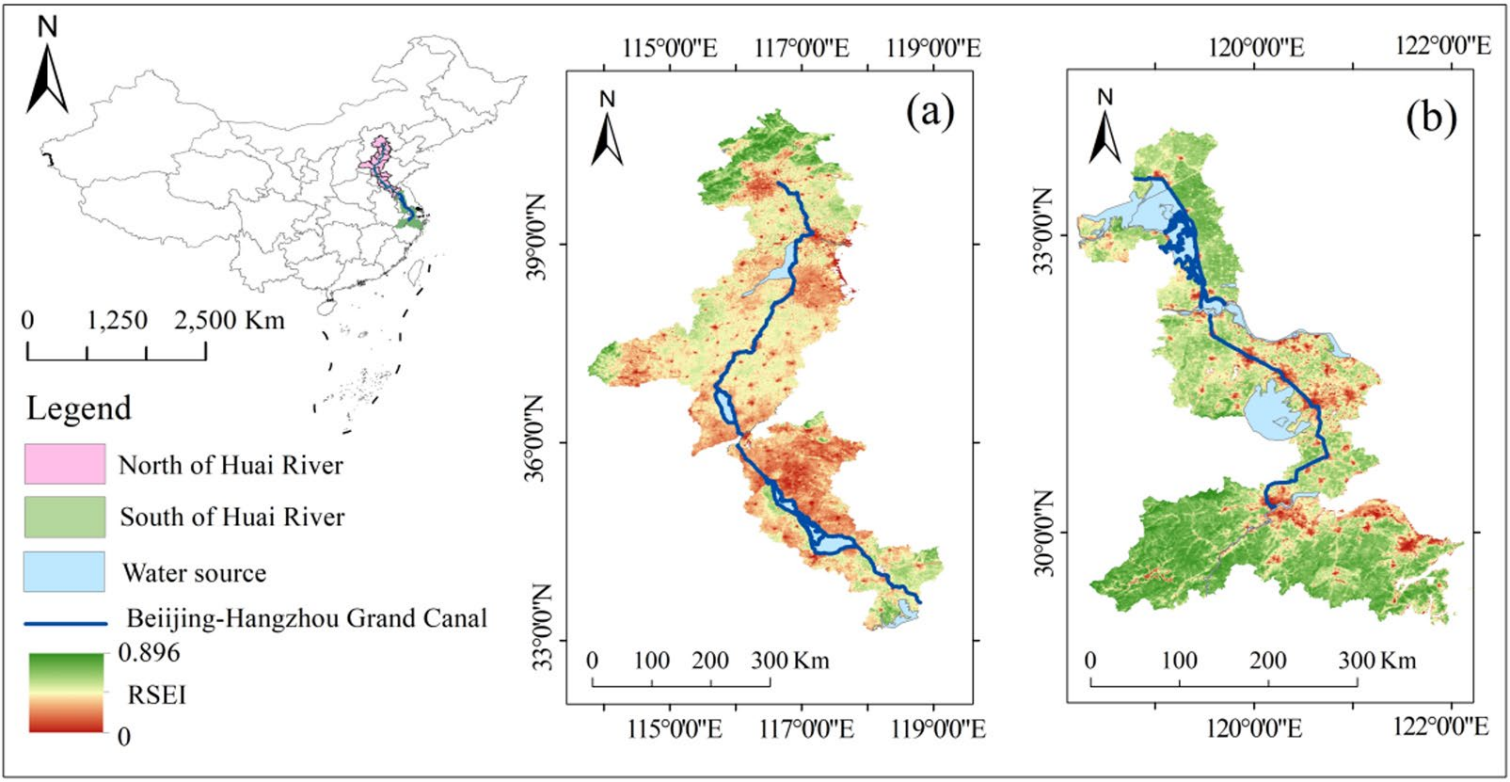
Location and RSEI distribution of the study area: (**a**) Section in the north of Huai River, (**b**) Section in the south of Huai River. The basemap was generated by the authors using Google Earth Engine (GEE, https://earthengine.google.com, accessed 2025) with MODIS satellite imagery (MOD09A1, MOD11A2, MOD13A1) from NASA LAADS DAAC (https://ladsweb.modaps.eosdis.nasa.gov). The Remote Sensing Ecological Index (RSEI) was calculated from growing-season (May–September) MODIS data (2000–2020) within a 5-km buffer zone along the canal. The map was created using GEE’s interactive visualization tools.

This study takes the Beijing-Hangzhou Grand Canal as the core research area (Fig. 1). It is the longest and largest ancient canal in the world, running through eastern China, with a total length of nearly 1,800 km. It flows through six provinces (municipalities directly under the Central Government), namely Beijing, Tianjin, Hebei, Shandong, Jiangsu and Zhejiang, forming a “golden waterway” connecting the north and south of China. It is not only a “living cultural heritage” that carries the history of Chinese civilization for thousands of years, but also an ecological corridor running through the core geographical units of China^27^. The evolution of its ecological environment profoundly reflects the complex interaction between natural and human elements.

The most distinctive geographical feature of the study area lies in its crossing of the iconic natural geographical dividing line between the Qinling-Huaihe Mountains. Based on this, we divides the Beijing-Hangzhou Grand Canal into two comparative units: the northern section (north of the Huai River) and the southern section (south of the Huai River). The northern section of the canal has a temperate monsoon climate. The average annual precipitation is generally below 800 millimeters, water resources are relatively scarce, and winters are cold. There is a freezing period in the river course, and the ecological background is relatively fragile. The southern section belongs to the subtropical monsoon climate, and the annual precipitation is mostly more than 800 to 1600 millimeters, a dense network of waterways, distinct four seasons, and no freezing period^14^. It enjoys more favorable water and heat conditions to support the ecosystem.

### Spatial gradient design and corridor zoning

To explicitly quantify the spatial gradient of ecological quality along the canal corridor, a buffer-based analytical framework was implemented, following the approach of Xiong and Jin^28^. First, a 5-km bilateral buffer zone was created along the central vector line of the Beijing-Hangzhou Grand Canal to define the extent of the proximal ecological corridor. All subsequent pixel-level analyses were constrained within this buffer. Second, to examine the gradient perpendicular to the canal, this buffer was further subdivided into contiguous 500-meter-wide distance belts. The statistical characteristics (mean, standard deviation) of the RSEI and its components were calculated for each belt within the northern and southern sections separately, enabling a detailed comparison of how ecological quality varies with distance from the canal.

### Datasets and preprocessing

The data for this study consist of two components, namely remote sensing imagery for RSEI calculation and a multi-source dataset of environmental and socioeconomic driving factors. All MODIS satellite data were accessed through the GEE cloud platform from the NASA LAADS website. Growing-season imagery (May to September) from 2000 to 2020 was selected to minimize phenological variability and focus on the period of maximum vegetation activity. MODIS products were scaled according to their respective metadata conversion coefficients and composited into annual growing-season means. The complete data sources are summarized in Table 1.

For the driving factor dataset, eight variables across four categories were compiled to support the XGBoost-SHAP attribution analysis. Topographic factors (DEM, slope, and aspect) were obtained from the European Space Agency’s Copernicus programme at 30 m resolution. Slope and aspect were derived from DEM within GEE using standard gradient algorithms and subsequently resampled to 500 m. As static physical features of the landscape, these variables were represented by a single time-invariant layer applied uniformly across all model years.

Climate factors (annual mean precipitation and annual mean temperature) and land use type were sourced from the Earth Resources Data Cloud platform. Socioeconomic factors (GDP, population density, and nighttime light intensity) were obtained from the Resource and Environment Science and Data Center of the Chinese Academy of Sciences. These driving factor datasets are available at five-year intervals (2000, 2005, 2010, 2015, and 2020). This temporal resolution reflects the standard publication cycle of China’s national gridded socioeconomic and land use datasets, which are produced at five-year intervals aligned with national census and land survey cycles. Although annual resolution would be preferable, the five-year interval nonetheless captures the dominant decadal trends in urbanization, land use change, and climatic shifts that are the primary focus of this attribution analysis. Each driving factor layer was matched to the corresponding RSEI year by nearest-year assignment, with the 2000, 2005, 2010, 2015, and 2020 layers applied to model the respective RSEI values of those years.

All datasets underwent systematic preprocessing to ensure spatial and temporal consistency. All input layers were resampled to a common 500 m resolution using bilinear interpolation and projected to the Albers Conic Equal Area coordinate system^14^. Climate and socioeconomic variables were spatially aggregated to 500 m resolution following resampling. All data were clipped to the 5 km bilateral buffer zone of the Beijing-Hangzhou Grand Canal, providing a spatially consistent data foundation for subsequent spatio-temporal analysis and machine learning modeling.

**Table 1:** Introduction to data sources.

Data classification	Data name	Time range	Spatial resolution	Source
MODIS data	MODO9A1-WET/NDBSI	2000–2020 May to September	500 m	National Aeronautics and Space Administration https://landsweb.modaps.eosdis.nasa.gov
	MOD11A2-LST		1 km	
	MOD13A1-NDVI		500 m	
Surface factor	DEM	Static	30 m	Copernicus panda website of the European Space Agency ( https://panda.coperni-cus.eu/panda)
	Slope		500 m	DEM-based data extraction
	Aspect		500 m	
Climate factor	Average annual temperature	2000,2005,2010,2015,2020	1 km	Earth Resources Data Cloud Website (http://www.gis5g.com)
	Annual precipitation	2000,2005,2010,2015,2020	1 km	Earth Resources Data Cloud Website (http://www.gis5g.com)
Human activity	Population density	2000,2005,2010,2015,2020	1 km	Resources and environment science data platform (https://www.resdc.cn/DOI/DOl.aspx?DOlid=32)
	Gross domestic product (GDP)	2000,2005,2010,2015,2020	1 km	Resources and environment science data platform (https://www.resdc.cn/DOI/DOl.aspx?DOlid=32)
	Land use type	2000,2005,2010,2015,2020	1 km	Earth Resources Data Cloud Website (http://www.gis5g.com)

### Research methods

1. RSEI Calculation Workflow.

The RSEI was constructed from four spectral indices representing key ecosystem components: greenness (Normalized Difference Vegetation Index (NDVI)), wetness (Wetness Index(WET)), heat (Land Surface Temperature(LST)), and dryness (Normalized Difference Bare Soil Index (NDBSI))^15,29,30^. The entire workflow was implemented on the Google Earth Engine (GEE) platform using MODIS imagery from the growing season (May to September) for each year from 2000 to 2020.

NDVI was computed from MODIS MOD13A2 product using the standard formula:

1 $$\:\mathrm{N}\mathrm{D}\mathrm{V}\mathrm{I}=\frac{{\mathrm{B}}_{2}-{\mathrm{B}}_{1}}{{\mathrm{B}}_{2}+{\mathrm{B}}_{1}}$$

where B1 and B2 are the red and near-infrared bands of MODIS, respectively. The NDVI values were scaled by 0.0001 as per MOD13A2 product specifications.

LST was obtained directly from the MOD11A2 product. The digital numbers were converted to Celsius using the following formula and then averaged over the growing season:

2 $$\:\mathrm{L}\mathrm{S}\mathrm{T}=\mathrm{D}\mathrm{N}\times\:0.02-273.15$$

3 $$\:\mathrm{S}\mathrm{I}=\frac{({\mathrm{B}}_{1}+{\mathrm{B}}_{6})-({\mathrm{B}}_{2}+{\mathrm{B}}_{3})}{({\mathrm{B}}_{1}+{\mathrm{B}}_{6})+({\mathrm{B}}_{2}+{\mathrm{B}}_{3})}$$

4 $$\:\mathrm{I}\mathrm{B}\mathrm{I}=\frac{2{\mathrm{B}}_{6}/({\mathrm{B}}_{2}+{\mathrm{B}}_{6})-\left[{\mathrm{B}}_{2}/({\mathrm{B}}_{1}+{\mathrm{B}}_{2})+{\mathrm{B}}_{4}/({\mathrm{B}}_{4}+{\mathrm{B}}_{6})\right]}{2{\mathrm{B}}_{6}/({\mathrm{B}}_{2}+{\mathrm{B}}_{6})+\left[{\mathrm{B}}_{2}/({\mathrm{B}}_{1}+{\mathrm{B}}_{2})+{\mathrm{B}}_{4}/({\mathrm{B}}_{4}+{\mathrm{B}}_{6})\right]}$$

5 $$\:\mathrm{N}\mathrm{D}\mathrm{B}\mathrm{S}\mathrm{I}=\frac{\mathrm{S}\mathrm{I}+\mathrm{I}\mathrm{B}\mathrm{I}}{2}$$

6 $$\:\mathrm{W}\mathrm{E}\mathrm{T}=0.1147{\mathrm{B}}_{1}+0.2489{\mathrm{B}}_{2}+0.2408{\mathrm{B}}_{3}+0.3132{\mathrm{B}}_{4}-0.3122{\mathrm{B}}_{5}-0.6416{\mathrm{B}}_{6}-0.5087{\mathrm{B}}_{7}$$

where B3 is blue band, B4 is green band, B5 is near-infrared band2, B6 is short-wave infrared band 1 and B7 is short-wave infrared band 2.

All four indices were normalized to a common scale [0,1] using min–max normalization prior to PCA to eliminate unit differences and ensure equal weighting. Principal Component Analysis was performed independently for each year (2000–2020) on the normalized four-dimensional datasets. The first principal component (PC1) was extracted from the eigenvalue decomposition of the covariance matrix^30^. To ensure the ecological interpretability of PC1, we examined the signs of the load coefficient of each component. NDVI and WET were expected to contribute positively, while LST and NDBSI were expected to contribute negatively. The variance explained by PC1 was computed as the ratio of the first eigenvalue to the sum of all eigenvalues, typically ranging from 70% to 85% across years, confirming that PC1 captured the dominant ecological pattern. The raw RSEI (PC1) was then linearly rescaled to the range [0,1] for intuitive interpretation:

7 $$\:RSEI=\frac{PC1-{PC1}_{min}}{{PC1}_{max}-{PC1}_{min}}$$

where values closer to 1 represent higher ecological quality. Finally, the RSEI values were classified into five ecological quality levels: poor (0-0.2), fair (0.2–0.4), moderate (0.4–0.6), good (0.6–0.8), and excellent (0.8-1.0) for spatial visualization and analysis. This equal-interval classification scheme follows the widely adopted standard in RSEI-based ecological assessments^15,21,31,32^, in which the normalized RSEI range of [0, 1] is divided into five equal intervals corresponding to progressively higher ecological quality.

(2) Kernel density estimation.

To reveal the intrinsic structure and pattern of the spatio-temporal evolution of regional ecological environment indicators (RSEI and its components) along the Canal from 2000 to 2020, this study adopted the Kernel Density Estimation (KDE) method^33^ for analyses. Unlike traditional time series graphs that can only reflect the trend of mean changes, KDE, as a non-parametric statistical method, can effectively estimate the probability density function of random variables, thereby revealing their distribution patterns, multimodal characteristics, and the concentration and dispersion trends over time. For a given sample of random variables, its kernel density estimation function is defined as:

8 $$\:f\left(x\right)=\frac{1}{nh}\sum\:_{i=1}^{n}k\left(\frac{x-{x}_{i}}{h}\right)$$

Among them, $\:{x}_{i}$ represents the observed values of samples with independent and uniform distribution (in this study, it refers to pixel-level indicators such as RSEI and NDVI). n represents the total sample size; K is the kernel function. This study employs the widely used Gaussian kernel function. h stands for Bandwidth, which is a key parameter for controlling the estimated smoothness. A suitable bandwidth can strike a balance between avoiding detail loss due to excessive smoothing and noise interference caused by undersmoothing^34^.

(3) Extreme gradient boosting and validation strategy.

Extreme Gradient Boosting (XGBoost) was employed in this study to model the complex relationships between RSEI and its driving factors along the Canal. As a gradient boosting decision tree algorithm, XGBoost excels at capturing nonlinear patterns and interaction effects through an iterative ensemble process, where successive trees correct residuals from previous ones^35^. Its advantages include built-in regularization to prevent overfitting and native handling of missing values, making it particularly suitable for ecological modeling. Model hyperparameters were optimized via grid search with cross-validation to ensure robust performance^24^.

This study employed the XGBoost regression model to elucidate the driving mechanisms of RSEI, implementing a systematic modeling and validation pipeline to ensure the reliability of the results. During the sample splitting stage, a spatial block splitting strategy was introduced to mitigate potential information leakage caused by spatial autocorrelation. The study area was first divided into uniform 1 km×1 km spatial grids, with each grid treated as a spatially dependent unit. Subsequently, the GroupShuffleSplit method (test_size = 0.2, random_state = 77) was applied to ensure that all pixels within the same grid were entirely isolated between the training and testing sets. Hyperparameter optimization was conducted using grid search combined with 5-fold cross-validation. The explored parameter space included: maximum tree depth (max_depth: [3,5,7,9]), learning rate (learning_rate: [0.01,0.05,0.1,0.3]), subsample ratio (subsample: [0.6,0.8,1.0]), feature subsample ratio (colsample_bytree: [0.6,0.8,1.0]), L1 regularization coefficient (reg_alpha: [0,0.5,1]), and L2 regularization coefficient (reg_lambda: [1,2,5]). To further enhance optimization stability, the grid search process was repeated three times. An early stopping mechanism (early_stopping_rounds = 50) was implemented during model training to prevent overfitting. Model performance was quantified using the root mean square error (RMSE) and the coefficient of determination (R^2^). To systematically identify and control spatial information leakage risks, this study concurrently calculated the spatial separation distance (based on nearest neighbor distances) between the training and testing sets, as well as the approximate Moran’s I index. These metrics were used to characterize spatial independence among samples and the strength of spatial autocorrelation, respectively, thereby ensuring methodological rigor in the model evaluation process.

(4) Shapley additive explanations.

To interrogate the decision logic of the XGBoost model, we adopted the SHAP framework, which is underpinned by cooperative game theory. This method calculates shapley values to characterize the marginal impact of each individual feature in shifting the model’s prediction from a predetermined baseline output^22^. This research investigated the SHAP framework across three dimensions. At the global level, features were prioritized by their mean absolute SHAP values. At the local level, individual predictions were explained. Furthermore, dependency plots were analyzed to identify both the directional trends and interaction effects of drivers on the RSEI.

**Fig. 2:**
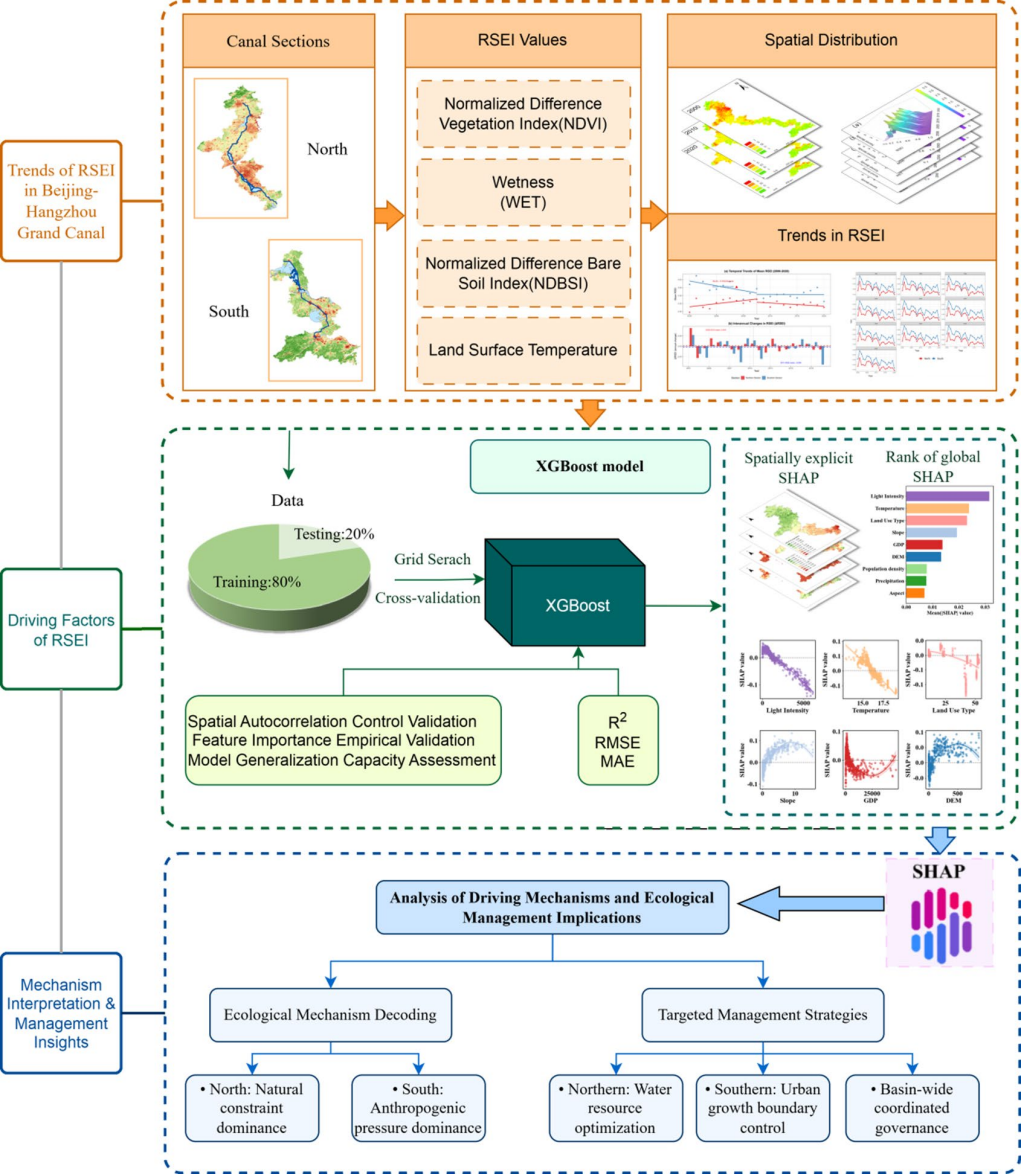
Framework of this study. The flowchart was created by the authors using Microsoft PowerPoint (Microsoft 365, https://www.microsoft.com/microsoft-365). All satellite data processing and RSEI calculation were performed on Google Earth Engine (GEE, https://earthengine.google.com) using MODIS products (MOD09A1, MOD11A2, MOD13A1) from NASA LAADS DAAC (2000–2020). The XGBoost-SHAP analysis was conducted in Python (version 3.9) using open-source libraries (XGBoost, SHAP). The basemap elements in the schematic are derived from GEE-processed MODIS imagery.

The integration of XGBoost and SHAP bridges the gap between model complexity and transparency, establishing a framework that excels in both predictive performance and feature interpretability. This approach not only identifies which factors are important but also elucidates how they influence ecological quality, thereby transforming complex statistical patterns into actionable scientific insights for environmental management. The framework of this study was shown in Fig. 2.

## Results

### XGBoost model performance and spatial validation

The performance metrics of the XGBoost models for both the northern and southern sections are summarized in Table 2. Under the spatial block splitting strategy, the testing set R^2^ reached 0.760 (RMSE = 0.064) for the northern section and 0.780 (RMSE = 0.064) for the southern section, indicating strong explanatory power of the models for RSEI spatial variability. Spatial analysis confirmed sufficient separation between the training and testing sets (minimum distance > 3.8 km) and moderate spatial autocorrelation levels (Moran’s I < 0.22), thereby ensuring the reliability of model evaluation. A comparative experiment further revealed that ignoring spatial autocorrelation would inflate model performance by approximately 3%, underscoring the necessity of the spatial block splitting approach.

**Table 2:** Performance metrics and spatial validation of XGBoost models for the northern and southern sections of the Beijing–Hangzhou Grand Canal.

Metric	Parameter	Northern section	Southern section
Training set performance	Number of samples	12,190	12,675
	RMSE	0.046	0.048
	R^2^	0.820	0.815
Testing set performance	Number of samples	3,041	3,182
	RMSE	0.064	0.064
	R^2^	0.760	0.780
Spatial validation	Minimum distance (km)	4.80	3.85
	Mean distance (km)	5.03	3.96
	Moran’s I (training)	0.100	0.119
	Moran’s I (testing)	0.172	0.216
Comparative experiment	R^2^ (spatial block)	0.760	0.780
	R^2^ (random split)	–	0.804
	Performance inflation	–	+ 0.024 (3.0%)

### Spatial distribution and north-south gradient of RSEI

**Fig. 3:**
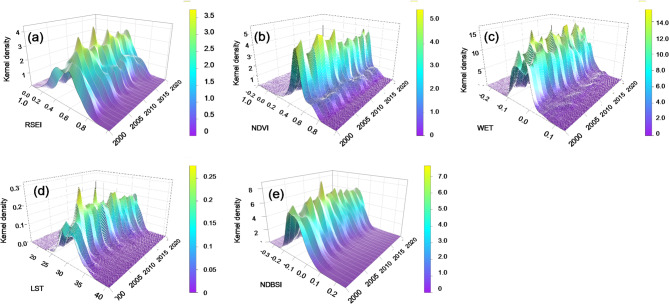
KDE of RSEI from 2000 to 2020 (**a** RSEI; **b** NDVI; **c** WET; **d** LST; **e** NDBSI).

The kernel density estimation of RSEI from 2000 to 2020 reveals a basin-wide improvement trend (Fig. 3). The peak density of RSEI fluctuates between 0.6 and 0.8 throughout the study period, with high-density areas shifting toward higher values after 2015. The early peaks are relatively scattered but become more concentrated in later years. NDVI peak values are mainly concentrated between 0.6 and 0.8, with high-density areas consistently moving toward higher values, a trend particularly pronounced after 2010. The WET index peaks remain concentrated between 0 and 0.1 with no clear directional trend, suggesting that humidity conditions along the Canal are primarily governed by seasonal and short-term climatic variability rather than long-term structural change. LST peaks are mainly concentrated between 25 and 35 °C without a sustained rising or falling tendency. NDBSI peaks, concentrated between − 0.1 and 0.1, show a slight upward shift especially after 2010, indicating a gradual increase in surface dryness.

**Fig. 4:**
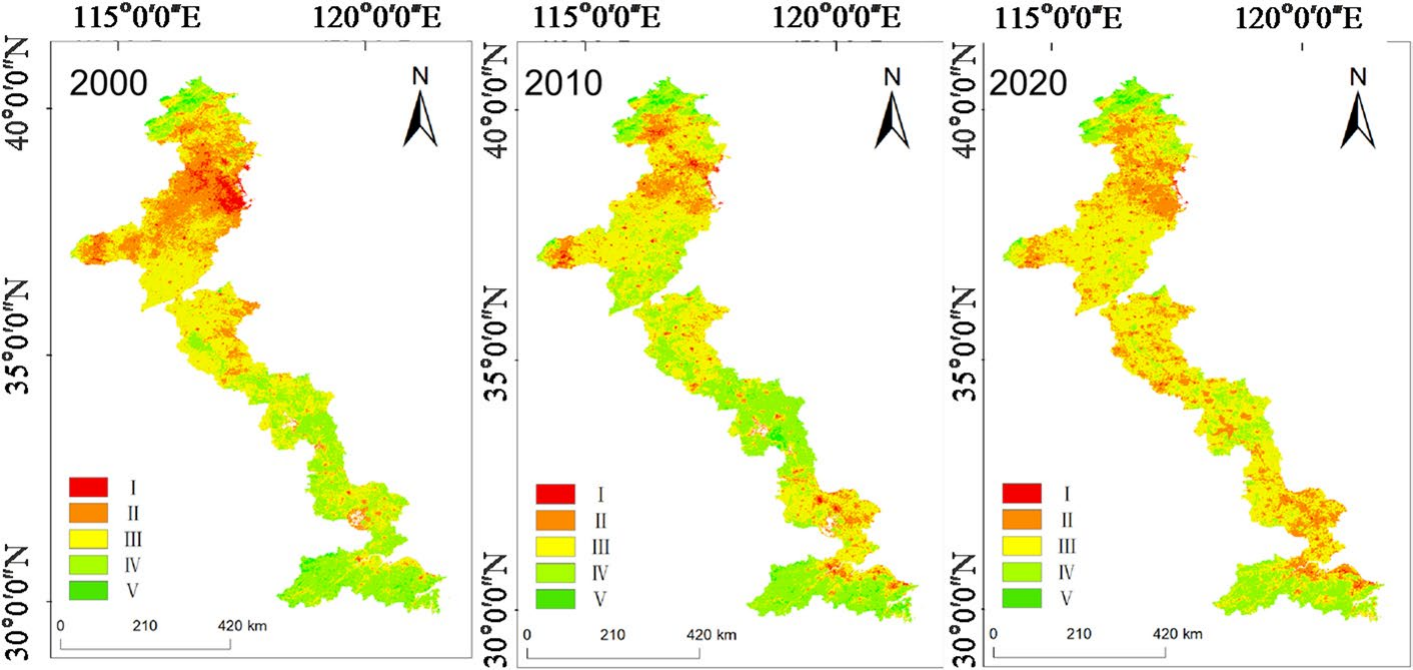
The spatial distribution map of RSEI from 2000 to 2020. The maps were generated by the authors using Google Earth Engine (GEE, https://earthengine.google.com) based on MODIS satellite data (MOD09A1, MOD11A2, MOD13A1) from NASA LAADS DAAC. RSEI was calculated for each growing season (May–September) and classified into five ecological quality levels: poor (0–0.2), fair (0.2–0.4), moderate (0.4–0.6), good (0.6–0.8), and excellent (0.8–1.0). The basemap imagery was accessed and visualized through GEE.

The spatial distribution of RSEI reveals a pronounced and persistent north-south gradient sustained across the entire 20-year study period (Fig. 4). The northern section, particularly from northern Shandong through Hebei, Tianjin, and Beijing, consistently exhibits low ecological quality corresponding to Grades I and II (poor to fair, RSEI < 0.4). The southern section, particularly southern Jiangsu and Zhejiang, maintains higher ecological quality corresponding to Grades IV and V (good to excellent, RSEI > 0.6). This gradient is quantitatively confirmed by the long-term mean RSEI values: the southern section  (0.580 ± 0.042) significantly exceeds the northern section.  (0.499 ± 0.023), with a mean difference of 0.081 (*p* < 0.001, Table 3). The northern section exhibited greater interannual stability, as reflected by its lower coefficient of variation (4.69% vs. 7.22% in the south), indicating more consistent environmental constraints. In contrast, the higher variability in the south reflects heightened sensitivity to both climatic fluctuations and intensive anthropogenic pressures.

**Fig. 5:**
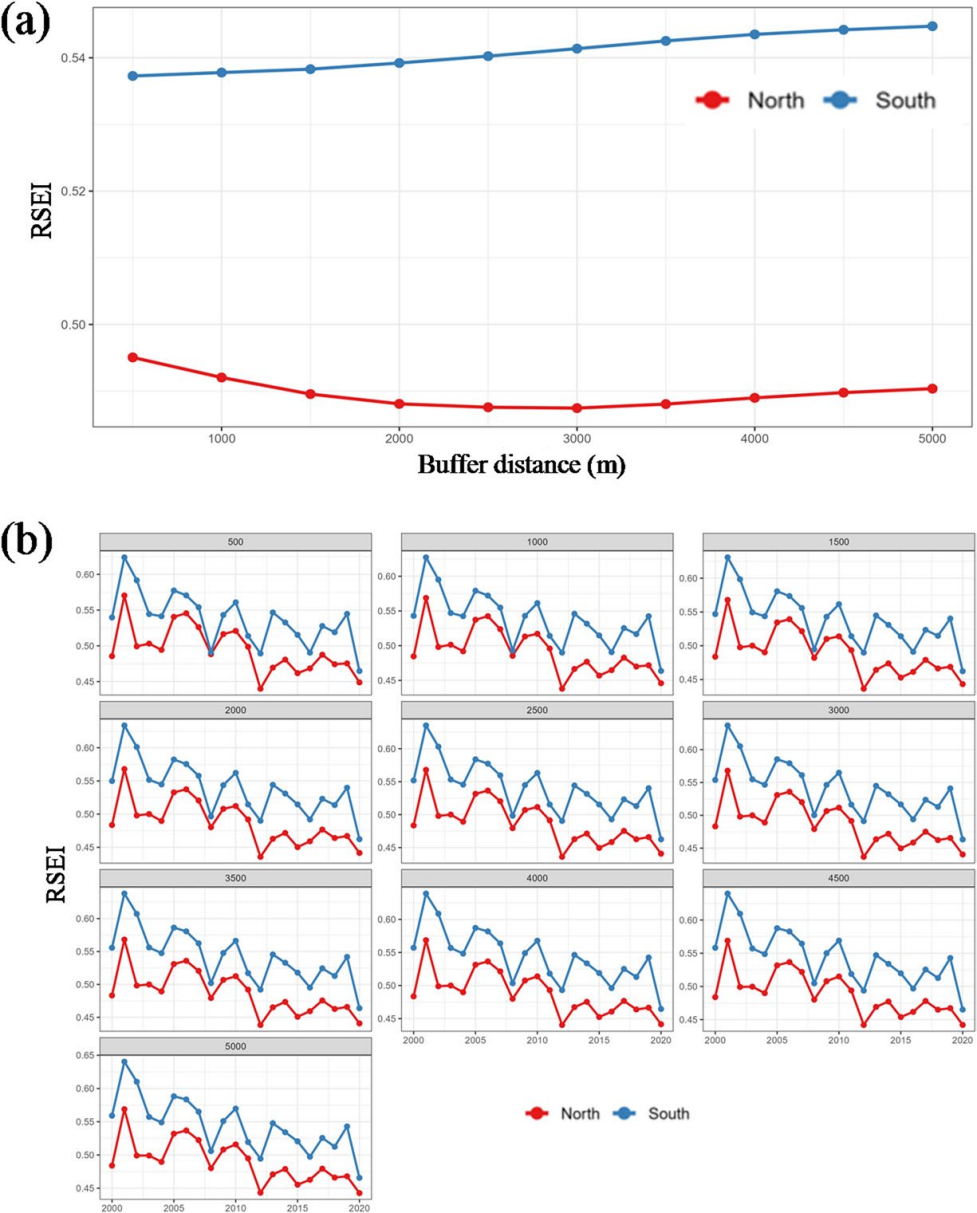
Spatial gradient and temporal dynamics of RSEI along the Beijing-Hangzhou Grand Canal. (**a**) Spatial gradient of mean RSEI along the buffer distance in the northern and southern sections; (**b**) interannual variations of mean RSEI in the northern and southern Sects.  (2000–2020).

Buffer zone analysis further characterizes the spatial gradient perpendicular to the canal corridor (Fig. 5). In the northern section, mean RSEI declines from 0.490 at 500 m to 0.460 at 2000 m and thereafter stabilizes between 0.448 and 0.455, suggesting that near-canal zones benefit marginally from riparian conditions but that the overall ecological level remains constrained by natural limitations. In contrast, the southern section displays a positive spatial gradient, with RSEI rising gradually from 0.538 at 500 m to 0.547 at 5000 m, indicating superior ecological conditions at greater distances from the canal where urbanization pressure is relatively lower.

### Driving factors of RSEI revealed by SHAP analysis

**Fig. 6:**
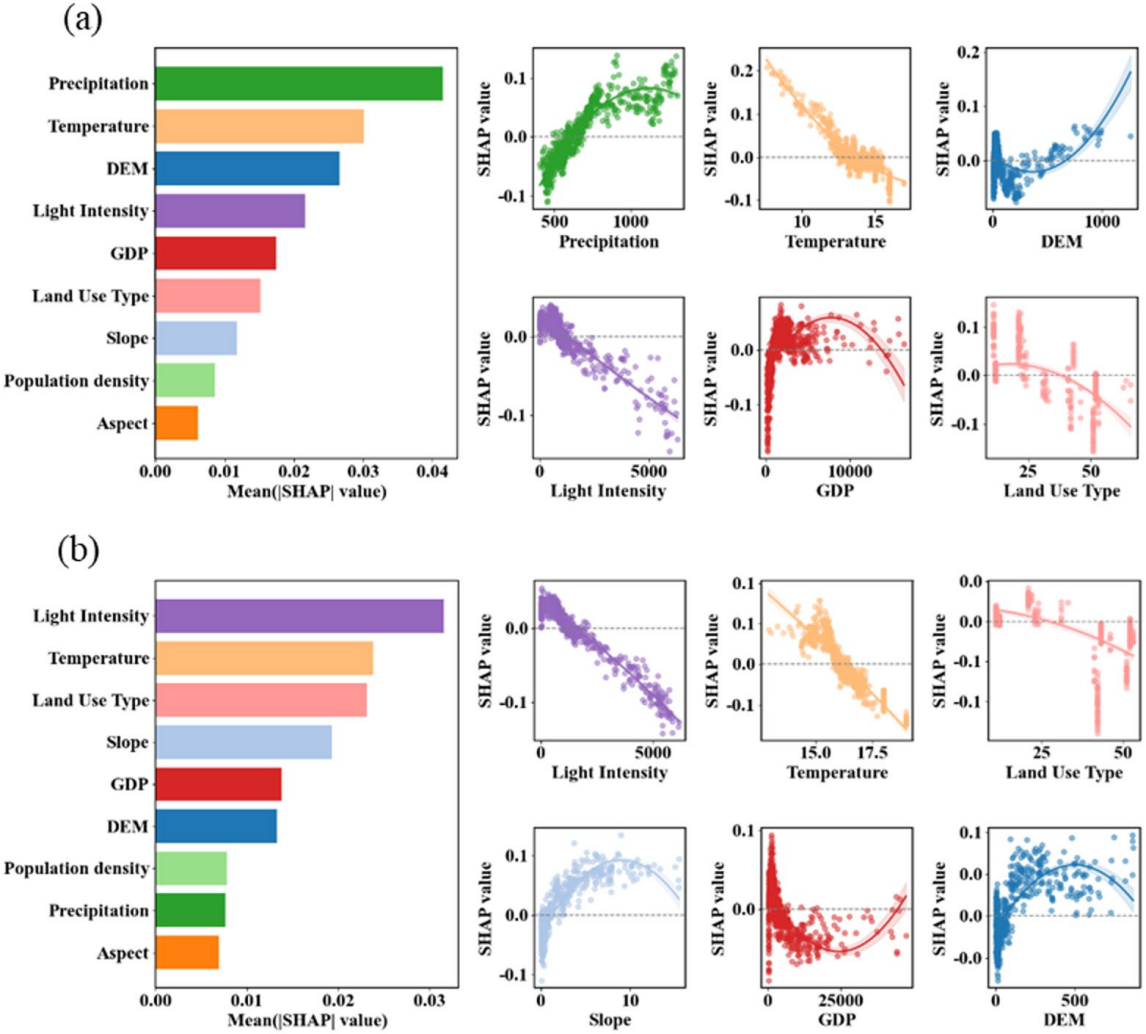
The global importance ranking of the SHAP of the driving factors in the northern (**a**) and southern (**b**) segments and the dependency relationship of the key factors.

The SHAP results for the northern section (Fig. 6a) revealed a distinct hierarchy of driving factors, with natural elements dominating the influence on RSEI. The main drivers of RSEI in the northern section were precipitation, temperature, DEM, night light intensity, and GDP in order of importance. Precipitation emerges as the most influential determinant, showing a consistently positive correlation with RSEI values across its measurement range. Temperature demonstrated a nonlinear relationship, characterized by an initial increasing effect that subsequently stabilizes. DEM exhibited elevation-dependent patterns, transitioning from positive to negative correlations along the elevation gradient. Both GDP and light intensity display minimal influence on RSEI variations, with their SHAP values remaining consistently near zero throughout their respective value ranges.

In contrast, the southern section (Fig. 6b) presented a fundamentally different driver hierarchy, where anthropogenic factors play a predominant role. The importance ranking of the driving factors in the southern section was night light intensity, temperature, land use type, slope and GDP. Light intensity stands out as the dominant driver, demonstrating a strong negative correlation with RSEI where increased luminosity corresponds to reduced ecological quality. Temperature maintained significant influence though with a different response pattern compared to the northern section. Land use type emerged as another important factor, showing clearly differentiated impacts across various categories. Slope exhibited a modest positive correlation, while other factors including GDP, precipitation, and DEM demonstrate comparatively limited effects on RSEI variations in this region.

**Fig. 7:**
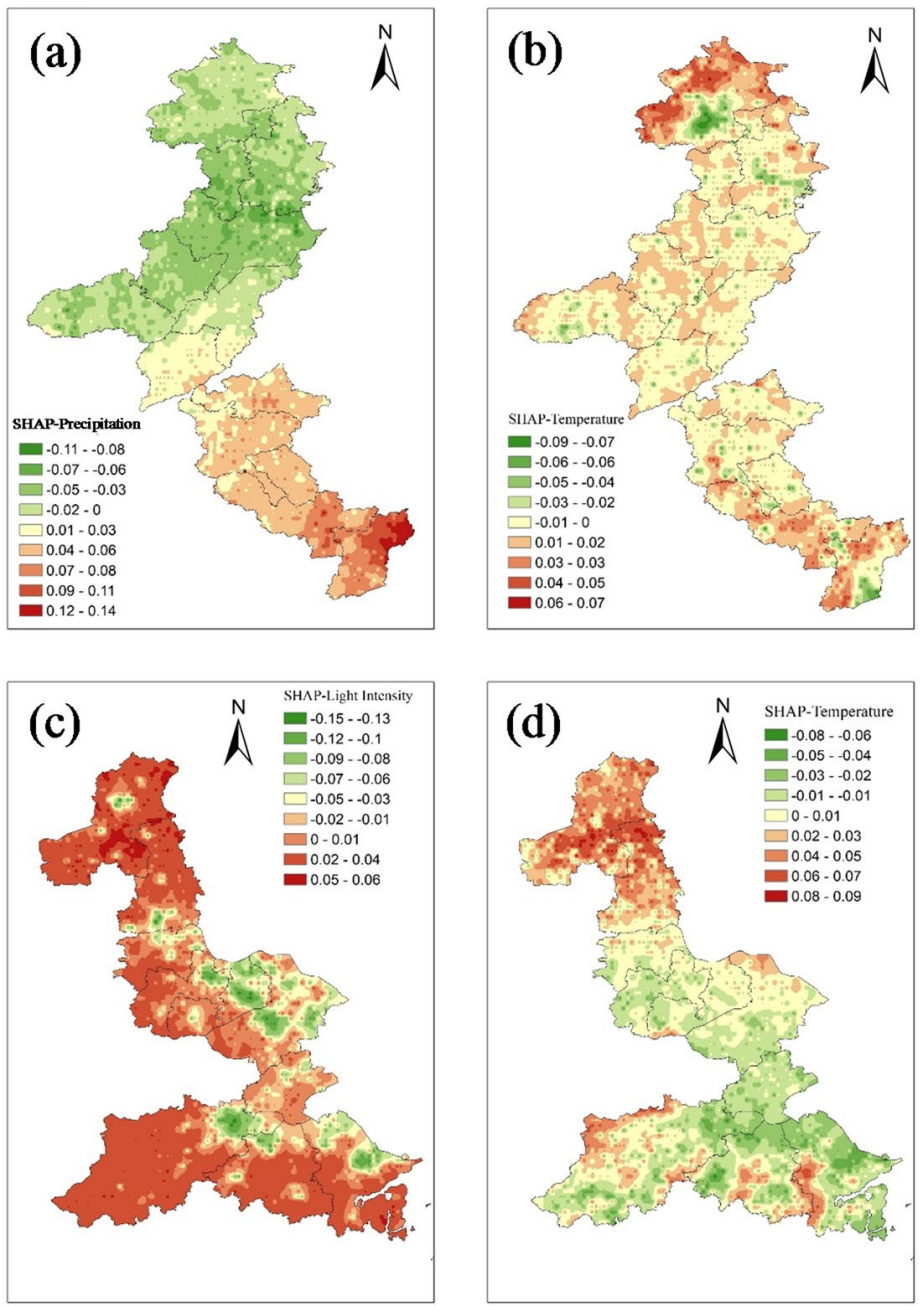
Spatially explicit SHAP values for key drivers in the northern and southern sections. (**a**) SHAP values for precipitation in the northern section; (**b**) SHAP values for temperature in the northern section; (**c**) SHAP values for nighttime light intensity in the southern section; (**d**) SHAP values for temperature in the southern section. The basemap was generated by the authors using Google Earth Engine (GEE, https://earthengine.google.com) with MODIS satellite imagery (MOD09A1) from NASA LAADS DAAC. SHAP values were calculated from the XGBoost model outputs and overlaid on the GEE-derived basemap using Python (matplotlib) and GEE visualization tools.

To visually elucidate the spatial heterogeneity of driver impacts and further substantiate the north-south mechanistic divergence identified in the global SHAP summary, we mapped the SHAP values of the two most influential factors in each section (Fig. 7). These spatially explicit representations reveal how the influence of key drivers varies across geographical space, providing a direct link between model interpretability and landscape-scale ecological patterns.

In the northern section, the SHAP map for precipitation (Fig. 7a) exhibits a pronounced spatial gradient, with high positive values (0.07–0.14) predominantly clustered in the eastern and southeastern buffer zones, where higher rainfall coincides with better-preserved wetlands, riparian vegetation, and irrigated farmlands. In contrast, the northwestern arid and semi-arid regions show near-zero or slightly negative SHAP values (− 0.11-0), underscoring water availability as the fundamental limiting factor for ecological quality. The SHAP distribution for temperature (Fig. 7b) displays a more mosaic pattern, with moderate positive values (0.03–0.07) scattered along vegetated corridors and agricultural plains where warming likely enhances growing-season productivity under adequate moisture conditions. Negative values (-0.09 to -0.03) are concentrated in urbanized patches and dry uplands, where temperature increases exacerbate water stress or surface heating. Notably, extensive areas exhibit near-neutral SHAP values (− 0.02–0.02), indicating that temperature acts as a secondary modulator rather than a primary driver in much of the northern corridor. Together, these maps reinforce the natural factor-dominated regime, where precipitation sets the broad ecological template and temperature fine-tunes local responses in a spatially heterogeneous manner.

In the southern section, the SHAP spatial pattern for nighttime light intensity (Fig. 7c) reveals a strong and spatially coherent anthropogenic signature. High negative SHAP values (-0.15 to -0.03) are densely concentrated in and around major urban clusters, particularly in the vicinity of Hangzhou, Suzhou, and Yangzhou. In these areas, intense nighttime illumination correlates directly with reduced RSEI. This visual evidence firmly links urban expansion and human activity intensity to ecological degradation. The SHAP map for temperature (Fig. 7d) shows a more diffuse and generally weaker influence, with values ranging from − 0.08 to 0.09. Slight positive contributions appear in rural and vegetated areas, whereas mild negative values occur in built-up zones. This pattern suggests that thermal effects are secondary to and often modulated by land-use change. These spatially explicit outputs validate the human activity-dominated mechanism, illustrating that the dominant driver (nighttime light) exerts its strongest suppressive influence precisely in zones undergoing intensive development, while natural factors such as temperature play a subordinate role.

### Temporal dynamics and trend analysis of RSEI

**Fig. 8:**
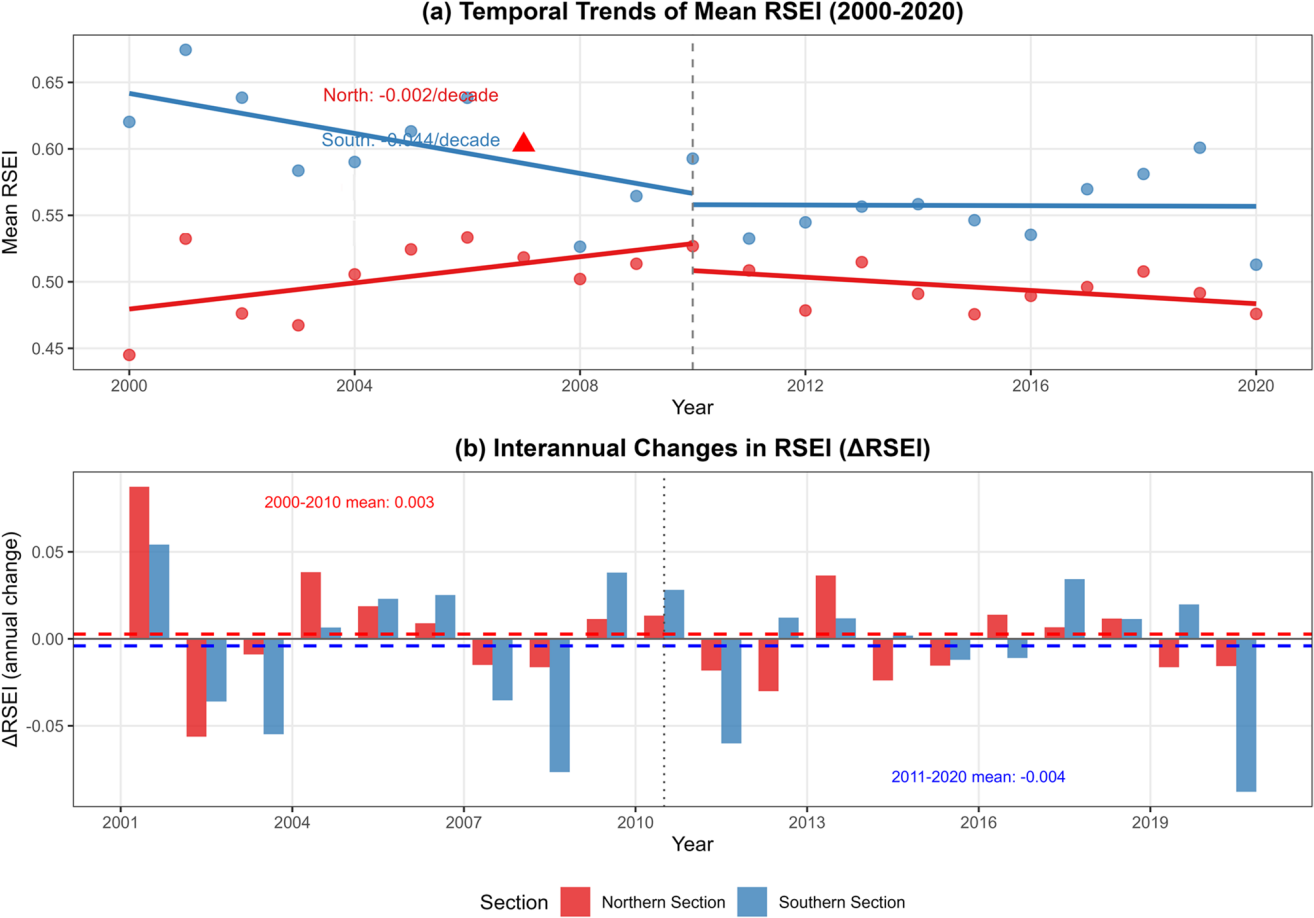
Temporal dynamics of RSEI along the Beijing-Hangzhou Grand Canal (2000–2020). (**a**) Temporal trends of mean RSEI, showing divergent patterns between northern (red) and southern (blue) sections. Dashed vertical line indicates 2010 segmentation point. (**b**) Interannual changes in RSEI (ΔRSEI), with positive values indicating improvement and negative values indicating degradation. The red triangle marks the significant change point detected in the southern section (2007).

Temporal analysis reveals fundamentally divergent trajectories between the northern and southern sections during 2000–2020, characterized by opposite long-term trends and distinct phase transitions (Fig. 8; Table 3). Overall, the southern section exhibits a significant declining trend, with Sen’s slope estimated at -0.045 per decade (*p* = 0.009) and linear regression yielding a consistent rate of -0.044 per decade (*p* = 0.001, R^2^ = 0.431). This decline is spatially extensive, with 73.4% of the southern corridor experiencing degradation and only 3.1% showing improvement over the study period. In contrast, the northern section shows no statistically significant long-term trend (Sen’s slope = -0.011 per decade, *p* = 0.381), but demonstrates a modest net improvement, with 44.3% of its area experiencing ecological enhancement and a change rate of + 0.015 per decade. Mann-Kendall analysis confirms a significant decreasing trend for the southern section ($\:\tau\:$= -0.419, *p* = 0.009) but no significant trend for the northern section ($\:\tau\:$= -0.143, *p* = 0.381).

Sub-period analysis reveals notable trend reversals around 2010 in both sections. During 2000–2010, the northern section showed a marginally positive trend (+ 0.049 per decade, *p* = 0.074), while the southern section experienced significant degradation (-0.075 per decade, *p* = 0.040). This pattern reversed in 2011–2020: the northern section shifted to a slightly negative trend (-0.013 per decade, *p* = 0.446), whereas the southern section exhibited a non-significant recovery signal (+ 0.022 per decade, *p* = 0.468). Pettitt’s test identified a statistically significant structural change point in the southern section in 2007 (*p* = 0.007), with no corresponding change point detected in the northern section, consistent with its more stable ecological baseline. Interannual variability analysis (Fig. 8b) further shows that negative ΔRSEI values in the southern section were concentrated during 2003–2007 and 2013–2015, periods coinciding with the most intensive phases of urbanization in the Yangtze River Delta. Together, these temporal patterns suggest a dynamic inversion of the Canal’s ecological trajectory, in which the historically superior south is undergoing accelerating degradation while the north maintains a relatively stable, if lower, ecological baseline.

**Table 3:** Statistical comparison of RSEI metrics between northern and southern Sects.  (2000–2020).

Metric	Northern section	Southern section	Difference	Statistical Test (*p*-value)
Mean RSEI (2000–2020)	0.499 ± 0.023	0.580 ± 0.042	0.081	< 0.001
Change rate (per decade)	0.015	− 0.054	− 0.01	–
Coefficient of variation	4.69	7.22	2.53	–
XGBoost model R^2^	0.76	0.78	+ 0.02	–
Improving area (%)	44.3	3.1	− 41.2	–
Degrading area (%)	21.6	73.4	51.8	–

### Model performance evaluation and validation

The performance of the XGBoost-SHAP framework was systematically evaluated through comparative analyses and rigorous validation procedures. As detailed in Table 4, the optimized XGBoost models demonstrated robust predictive capabilities for both northern and southern sections, achieving test set R^2^ values of 0.76 (northern) and 0.78 (southern) with corresponding RMSE of 0.064. These results substantially outperformed conventional methods including multiple linear regression (R^2^ = 0.58, RMSE = 0.121), confirming the superiority of machine learning approaches in capturing complex nonlinear relationships inherent in ecosystem responses. The marginal performance gap between training and testing sets (ΔR^2^ < 0.07) indicates effective overfitting control through regularization and cross-validation. Comparative analysis revealed XGBoost’s consistent superiority over alternative machine learning approaches, including Random Forest (ΔR^2^ = +0.02) and Support Vector Regression (ΔR^2^ = +0.08). This performance advantage aligns with findings in similar ecological machine learning applications where XGBoost’s gradient boosting architecture effectively handles complex environmental relationships^36^. Validation procedures further substantiated the framework’s reliability and interpretability. Feature ablation analysis demonstrated that removing SHAP-identified key drivers—precipitation in the north and nighttime light intensity in the south—caused substantial performance degradation (R^2^ reductions of 0.18 and 0.21, respectively), empirically confirming their causal importance rather than mere statistical association. Conversely, excluding low-importance variables yielded negligible effects (< 0.02 R^2^ change). These comprehensive evaluations establish that the integrated GEE-XGBoost-SHAP framework not only achieves superior predictive accuracy but also provides reliable, interpretable insights into the differential driving mechanisms operating along the Grand Canal’s north-south continuum.

**Table 4:** Comprehensive evaluation of modeling approaches for RSEI prediction.

Model/method	Dataset/comparison	*R* ^2^	RMSE	MAE
XGBoost (northern)	Training	0.82	0.046	0.053
XGBoost (northern)	Testing	0.76	0.064	0.062
XGBoost (southern)	Training	0.82	0.048	0.049
XGBoost (southern)	Testing	0.78	0.064	0.057
Multiple linear regression	Full dataset	0.58	0.121	0.094
Random forest	Testing (comparison)	0.74	0.089	0.066
Support vector regression	Testing (comparison)	0.68	0.106	0.082
Feature ablation (north)	Excluding precipitation	0.58	0.117	0.088
Feature ablation (south)	Excluding nighttime light	0.60	0.120	0.091

## Discussion

### Methodological advancements in earth observation for linear heritage monitoring

The integrated GEE-RSEI-XGBoost-SHAP framework developed in this study not only advances technical capability but also generates substantive new knowledge that previous methodological approaches were structurally unable to produce. Three dimensions of advancement merit specific discussion in light of the empirical results obtained.

First, the full-basin, long-term analytical scope enabled by GEE revealed a macro-ecological pattern that localized studies are inherently incapable of detecting. A persistent and statistically significant north-south RSEI gradient (mean difference = 0.081, *p* < 0.001) was sustained over two decades. Zhang et al.^12^ and Xie et al.^13^ confined to the Suzhou section and Beijing segment respectively, documented localized habitat degradation and water quality improvement, but could not determine whether these local trends were representative of or divergent from basin-wide dynamics. Our full-corridor analysis reveals precisely this distinction. The southern section’s degradation trajectory affects 73.4% of the southern corridor, while the northern section followed an opposite trend of modest improvement (+ 0.015 per decade). This basin-wide perspective transforms isolated observations into a coherent ecological narrative, demonstrating that methodological scale is not merely a technical parameter but a determinant of scientific conclusions.

Second, the multi-component RSEI framework exposed compound ecological stress dynamics that single-indicator approaches systematically miss. Our results show that while NDVI improved across both sections, consistent with the vegetation greening trends reported by Jiang et al.^14^, simultaneous increases in NDBSI and persistent LST levels partially offset these gains, particularly in the northern section. This compound pattern explains why overall RSEI improvement in the north remained modest (+ 0.015 per decade) despite visible vegetation recovery. The RSEI’s capacity to integrate competing ecological signals prevents the overestimation of ecological improvement that a vegetation-only index would produce. This finding has direct implications for ecological assessment methodology. In regions where greening programs are actively implemented, single-indicator monitoring risks generating misleadingly optimistic evaluations of ecosystem health.

Third, and most critically, the XGBoost-SHAP framework uncovered driver-response relationships that are both quantitatively precise and ecologically interpretable in ways that conventional methods cannot achieve. Geographic detectors and regression models employed in prior Grand Canal studies^15^ can rank factor importance but cannot reveal the shape, directionality, or conditional nature of these relationships. Our SHAP analysis produced three specific advances. It quantified that removing precipitation from the northern model caused an R^2^ reduction of 0.18, empirically confirming its causal primacy rather than merely inferring it. It further revealed that nighttime light intensity’s negative effect on southern RSEI is non-linear, with suppression effects intensifying sharply above a luminosity threshold corresponding to high-density urban zones around Hangzhou, Suzhou, and Yangzhou. It also demonstrated that temperature’s influence differs fundamentally between sections. In the northern section, temperature acts as a growth facilitator, whereas in the urban south it functions as a stress amplifier. This context-dependency would be averaged into statistical insignificance by linear models. These findings do not simply validate known patterns; they resolve previously unquantifiable questions about where, how, and under what conditions each driver shapes ecological quality along the Canal.

### Mechanistic insights into north-south ecological divergence

In the northern section, the natural factor-dominated mechanism is consistent with its temperate semi-humid/semi-arid climatic context. The stable positive correlation between precipitation and RSEI confirms water resources as the fundamental ecological bottleneck, corroborating the primacy of precipitation identified in other arid regions^37^. Critically, our analysis moves beyond conventional correlation analyses by revealing a nonlinear influence of temperature through SHAP dependence plots. This pattern suggests complex physiological thresholds in vegetation response that linear models might oversimplify^30^. The minimal influence of socioeconomic factors such as GDP and nighttime light intensity, with SHAP values clustered near zero, further highlights how strong natural constraints can mask the signal of human activity. This represents a nuance difficult to capture with traditional methods.

Conversely, the southern section’s human activity-driven mechanism stems from the tension between its superior natural endowment and intensive development. The markedly negative correlation between nighttime light intensity and RSEI establishes urban expansion as the predominant stressor, a finding that aligns with but quantitatively refines earlier observations of socioeconomic dominance in developed regions^38^. Through SHAP dependence analysis, we further elucidate how this anthropogenic pressure operates - urban land conversion synergistically undermines ecosystem integrity through both direct habitat replacement and indirect microclimatic alterations. This mechanistic understanding advances beyond the statistical correlations typically obtained through conventional linear approaches^39^. Particularly noteworthy is the prominent role of land use type among core drivers, where our SHAP results not only confirm its established importance in ecological studies^15^ but also delineate its specific categorical impacts, revealing how the transition between natural vegetation and built-up areas directly modulates ecosystem service functions. The emergence of this detailed mechanistic pathway, captured through our interpretable framework, demonstrates how human modifications of land surface characteristics have become the primary force reconfiguring the ecological pattern in this naturally advantaged region.

While the XGBoost-SHAP framework successfully identified climate factors and human activities as the primary determinants of north-south RSEI divergence, several additional factors may also contribute to the observed spatial heterogeneity and warrant further discussion. First, soil characteristics differ substantially between the two sections. The northern section contains extensive saline and alkaline soils with degraded structure, reduced organic matter content, and lower water retention capacity, which structurally constrain vegetation productivity independent of climate inputs^40,41^. Second, although DEM was included as a predictor variable, the underlying geomorphological context generates distinct local microclimatic regimes beyond what elevation alone captures. Studies on RSEI drivers in similar basins have confirmed that elevation affects the water-heat combination and soil type, resulting in vertical differentiation of vegetation habitats and ultimately contributing to spatial heterogeneity in ecological quality^42^. Third, hydrological connectivity represents an underexplored spatial driver: the dense, historically maintained water network of the Jiangnan region in the southern section inherently elevates the WET component of RSEI, a pattern shaped by centuries of hydraulic engineering as much as by contemporary precipitation, and prior research in the Taihu Lake Basin confirms that land-use type, closely tied to this water network structure, is the dominant driver of RSEI spatial heterogeneity in the region, explaining nearly 60% of the variance^43^. Fourth, historical land-use legacies in the rapidly developed Yangtze River Delta may impose persistent ecological deficits not fully captured by current-period anthropogenic indicators such as nighttime light intensity. Research confirms that continuous urban expansion in the region has exerted a significant negative spatial spillover effect on ecosystem health, with land urbanization increases leading to measurable declines in ecological quality even beyond local boundaries^44^. Finally, differences in vegetation community structure and biodiversity between the subtropical south and temperate north confer differential ecosystem resilience to disturbance, which may partly explain why the southern section, despite its superior baseline, exhibits greater sensitivity to anthropogenic pressures. The subtropical humid region shows the highest human-activity-related contribution to vegetation change in China (89.4%), while the temperate humid zone exhibits a more balanced response between climate and human activity (55.8%), consistent with the north-south mechanistic divergence identified in this study^45^. Future research integrating soil databases, fine-resolution hydrological network data, and biodiversity inventories would help disentangle these compounding influences.

Beyond spatial heterogeneity, two temporal inflection points identified in the results, around 2010 and post-2015, warrant explicit mechanistic interpretation, as they reflect the imprint of national policy transitions on the Canal’s ecological trajectory. The year 2010 represents a structural turning point in China’s environmental governance: the 12th Five-Year Plan (2011–2015) introduced binding targets for energy intensity reduction and pollutant emission caps, which began to constrain the rapid urban expansion that had driven the significant RSEI decline in the southern section during 2000-2010^14,17^. Crucially, this period also coincided with the initiation of the South-to-North Water Diversion Project’s Eastern Route along the Grand Canal corridor, which began supplying water to Shandong in November 2013 and subsequently augmented water availability across the northern section^46^, providing a plausible mechanism for the modest ecological stabilization observed in the northern section after 2010. The post-2015 shift toward higher RSEI values-reflected in the rightward migration of the kernel density peak and the more concentrated NDVI distribution-aligns with the implementation of the 13th Five-Year Plan (2016–2020) and, critically, the elevation of the Grand Canal Cultural Belt to a national strategic initiative in 2017^42^. This policy mandate explicitly integrated ecological restoration with heritage conservation along the entire corridor, triggering large-scale afforestation programs, wetland rehabilitation projects, and stricter riverside construction controls. These findings underscore that the Canal’s ecological trajectory is profoundly shaped by punctuated policy interventions-a dimension that carries direct practical implications for heritage corridor management worldwide.

### Transferability of the analytical framework to global linear systems

The core value of the GEE data platform + RSEI evaluation framework + XGBoost-SHAP-driven parsing technology chain constructed by this study lies in its high modularity, scalability and portability. This provides a proven toolbox for addressing similar monitoring and management challenges faced by numerous large-scale linear cultural heritages and ecological corridors around the world.

The cloud processing mode based on GEE is the technical cornerstone for the method to be scalable. It addresses the pain points in traditional remote sensing processing, such as difficult data acquisition, high computational costs, and fragmented analysis processes. Whether the research subjects are the Danube in Europe, the Nile in Africa, or the Erie Canal in the Americas, researchers can utilize the long-term satellite data archive with global coverage in GEE to reproduce the full-basin time series analysis process of this study and achieve rapid deployment of monitoring capabilities. Tamiminia et al.^19^systematically reviewed GEE applications across diverse global environments and confirmed the platform’s proven capacity for large-scale, multi-sensor ecological monitoring across Asia, North America, Africa, and Europe Frontiers, providing the technical foundation for extending our framework to heritage corridors beyond China.

The conceptual model of the relative importance of the nature-human driving force changing with the environmental gradient is equally universal. This study finds that in the north where water resources are limited, natural factors dominate, while in the south where development pressure is intense, human factors play a dominant role. This gradient-driven shift closely parallels findings from comparable studies in other regions of China. Li et al.^47^ applied GEE and SHAP-based interpretable machine learning to evaluate ecological quality in three arid irrigation oases in China and confirmed that precipitation was the primary driver, with SHAP values ranging from 0.031 to 0.30, and identified a critical threshold at approximately 164 mm/yr beyond which ecosystem suppression transitions to enhancement Frontiers, a regime-shift pattern directly analogous to the natural-factor-dominated mechanism identified in the northern section of this study. This rule is highly likely to recur in other linear systems spanning significant environmental or development gradients. For instance, in the Suez Canal, the driving mechanism might shift from being climate-dominated in the arid desert section to being dominated by shipping activities and coastal development toward the Mediterranean coast. In the rainforest section of the Panama Canal, the interplay between biodiversity conservation and shipping impact may be the core driving relationship. The framework of this study provides a ready-made analytical method for testing these hypotheses.

### Limitations and future research directions

While this study advances the understanding of ecological dynamics along the Beijing-Hangzhou Grand Canal, several limitations should be explicitly acknowledged across three dimensions. Regarding the methodological framework itself, the XGBoost-SHAP approach, despite its interpretability advantages, identifies statistical associations rather than proven causal mechanisms. As explicitly noted in analogous XGBoost-SHAP applications to urban ecological resilience, this framework is fundamentally data-driven and does not explicitly establish causal mechanisms; the reported relationships should therefore be interpreted as model-based associations rather than direct causal evidence PubMed Central^47^. The spatial block cross-validation strategy effectively mitigates spatial autocorrelation (Moran’s I < 0.22), yet cannot fully eliminate it in a linear corridor where spatial dependence may extend beyond the separation distance of 3.8 to 4.8 km achieved in this study. Furthermore, SHAP values quantify each feature’s marginal contribution within the model’s learned function. This contribution is conditioned on the model’s training data distribution, and extrapolation to ecological conditions outside the observed range, such as future climate scenarios, should therefore be treated with caution. The R^2^ values of 0.76 to 0.78, while substantially outperforming conventional methods, also indicate that approximately 22 to 24% of RSEI spatial variance remains unexplained. This suggests that important drivers may still be absent from the current feature set.

Regarding the selection of influencing factors, the current eight-variable framework covers the major categories of natural and anthropogenic drivers but omits several potentially important factors. Shipping intensity and vessel traffic are defining characteristics of the Grand Canal as a functioning waterway. They introduce pollution loads and bank erosion dynamics that are not captured by any proxy in the current variable set. Water quality parameters such as nitrogen and phosphorus concentrations directly affect aquatic and riparian ecosystems and are similarly absent from the analysis. At the land-atmosphere interface, aerosol optical depth and PM_2.5_ data are not included as drivers. The documented air pollution gradient along the Canal corridor^10,11^ may therefore confound the interpretation of temperature and nighttime light effects. Additionally, the five-year temporal resolution of socioeconomic variables such as GDP and population density may smooth over short-term economic shocks. Events such as the 2008 financial crisis and post-2015 industrial restructuring could explain some of the interannual RSEI variability observed in the results but cannot be captured at this temporal granularity.

Regarding temporal dynamics, while this study identifies 2010 and 2015 as important temporal inflection points and proposes policy-based explanations, the current modeling framework is cross-sectional rather than spatiotemporal. It therefore cannot formally attribute observed trend changes to specific policy interventions. Future research should adopt spatiotemporal models such as Geographically and Temporally Weighted Regression (GTWR) or panel data approaches to disentangle policy effects from climatic variability^14,48,49^. Integrating process-based ecological models would further help to establish causal mechanisms beyond the statistical correlations identified here. Incorporating finer temporal resolution socioeconomic data would additionally improve the detection of short-term human activity impacts on ecological quality.

## Conclusion

This study employed the Google Earth Engine platform and an interpretable XGBoost-SHAP framework to analyze the spatiotemporal dynamics and driving factors of ecological quality along the Beijing-Hangzhou Grand Canal from 2000 to 2020. The main conclusions are as follows:

1. A sustained north-south spatial gradient in ecological quality was identified, with southern sections consistently exhibiting higher RSEI values (mean = 0.580 ± 0.042) compared to northern sections (mean = 0.499 ± 0.023) throughout the study period. Temporally, the northern section showed modest improvement (+ 0.015/decade) with 44.3% of areas experiencing ecological enhancement, whereas the southern section displayed significant degradation (-0.054/decade, *p* = 0.009) with 73.4% of areas deteriorating despite its superior baseline condition.
2. The XGBoost-SHAP analysis revealed fundamentally different driving mechanisms between sections. The northern section exhibits a “natural factor-dominated” regime, where ecological quality is primarily controlled by precipitation and temperature, reflecting strong climatic constraints on ecosystem functioning in this water-limited region. In contrast, the southern section demonstrates a “human activity-dominated” regime, with nighttime light intensity and land use change emerging as the principal drivers, indicating that urbanization pressure has become the predominant force reshaping ecological patterns in this naturally advantaged region.
3. The integrated GEE-XGBoost-SHAP framework successfully addresses the limitations of conventional linear models in capturing complex nonlinear interactions inherent in coupled human-natural systems. This methodological advancement not only achieved superior predictive performance (R^2^ = 0.76–0.78) but also provided interpretable, quantitative attribution of driver contributions, transforming ecological assessment from descriptive pattern recognition to mechanistic understanding.
4. This research provides a scientific basis for spatially differentiated ecological management strategies along the Grand Canal—prioritizing water resource management and climate adaptation in the northern section while emphasizing urban growth control and pollution mitigation in the southern section. The transferable analytical framework offers valuable methodological insights for the conservation and monitoring of large-scale linear cultural heritage sites and ecological corridors worldwide.

## Data Availability

All data supporting the findings of this study are publicly accessible. The remote sensing datasets, including MODIS surface reflectance (MOD09A1) and land surface temperature (MOD11A2) products, were obtained through the Google Earth Engine cloud platform (https://earthengine.google.com) and NASA’s LAADS DAAC (https://ladsweb.modaps.eosdis.nasa.gov). Digital Elevation Model data were sourced from the European Space Agency’s Copernicus programme (https://panda.copernicus.eu). Climate variables (annual precipitation and temperature), land use/land cover data, and socioeconomic datasets (gridded GDP, population density, and NPP-VIIRS nighttime light imagery) are all available from the Resource and Environment Science and Data Center, Chinese Academy of Sciences (https://www.resdc.cn).

## Code availability

The computational code developed for this study has been made openly available to ensure full reproducibility. The Google Earth Engine JavaScript scripts used for calculating the Remote Sensing Ecological Index (RSEI), including all component derivations and integration, are accessible online. The complete analytical pipeline for the XGBoost-SHAP framework is hosted in a GitHub repository (https://github.com/bzysir/Beijing-Hangzhou-Grand-Canal) under an open-source license. This repository contains all necessary code for data preprocessing, model training, SHAP value computation, and visualization, facilitating validation and reuse by the research community.
